# A dose-escalation clinical trial to evaluate the safety and immunogenicity of a replication-defective HIV-1 vaccine-HIVAX

**DOI:** 10.1186/1742-4690-9-S2-P127

**Published:** 2012-09-13

**Authors:** F Tung, J Tung, M Fischl

**Affiliations:** 1 GeneCure Biotechnologies, Norcross, GA, USA; 2 University of Miami, Miami, FL, USA

## Background

Replication-defective SIV elicited protective immunity in animals. In this first-in-human therapeutic vaccination study, a replication-defective HIV-1 vaccine was tested in HIV-1 infected subjects under antiretroviral therapy.

## Methods

A010 is an ongoing randomized, placebo-controlled dose-escalation clinical trial to evaluate the safety and the immunogenicity of two doses of a replication defective HIV-1 vaccine (HIVAX™) in subjects receiving stable highly active antiretroviral therapy (HAART) who have an HIV-1 RNA <50 copies/ml and CD4 cell count >500 cells/mm3. Following the randomized placebo-controlled vaccination phase subjects who received active vaccine and who meet eligibility will undergo a 12-week analytical antiretroviral treatment interruption.

## Results

HIVAX™ is well tolerated in HIV infected subjects. Only mild injection site reaction occurred with transient duration. No medical treatment is necessary. High level of cell-mediated immune responses measured by ELISPOT assay was noticed after vaccination.

## Conclusion

The replication defective HIV vaccine appears no severe adverse effect in HIV-1 infected subjects. High level of cell-mediated immune response was elicited in the vaccinees. HIVAX™ is worth for further evaluation of protective efficacy.

